# Medical and Philosophical Intelligence

**Published:** 1816-09

**Authors:** 


### MEDICAL AND PHILOSOPHICAL INTELLIGENCE.

*ROYAL SOCIETY.*—On Thursday, June 27, a paper, by Sir Everard Home, was read, giving a further account of the mechanism by means of which insects can walk contrary to gravity, as up perpendicular walls, or along the roofs of rooms. Since his first paper was read, Mr. Bower has drawn the apparatus of the feet of flies, determined by means of the microscope. It turns out different from what he formerly described. In the common bottle-fly, every toe is supplied with two suckers, very similar in appearance to the pieces of wet leather and string with which boys lift up stones. These suckers appear to be put in action by the voluntary muscles of the insect. In the horse-fly, each toe is supplied with three suckers. These suckers may be seen by means of a glass, when a common fly is walking along the inside of a tumbler. The author concluded his paper with the description of certain cushions upon the feet of some insects, to take off the effect of a sudden change from a state of rapid motion to a state of rest.

At the same meeting, part of a paper, by Dr. Bernardino Antonio Gomez, Physician to the Prince Regent of Portugal, on the Mode of Fumigating Infected Letters, was read. Letters from places supposed to be visited by the plague are fumigated with vinegar, after being cut in several places, before they are delivered to the persons to whom they are directed. It became a question in Portugal whether this mode of fumigation was sufficient. Government gave orders to substitute Morveau's mode, by fumigating with chlorine. The author of the paper was doubtful how far this practice would be effectual, unless the letters were opened. He requested of government to be allowed to investigate the point, in the first place, experimentally, and his request was granted. He found that letters, or paper parcels, exposed to the fumes of chlorine, even though completely closed by means of sealing wax, are penetrated by it, and retain the smell for several days. He impregnated tow, cotton, silk, wool, and fur, with the vapour of putrid meat, inclosed a portion of each in a letter, and exposed the letters letters to the fume of chlorine for half an hour. The putrid odour was destroyed in the tow and cotton; the silk retained a little of it; and the wool and fur smelled still very strongly of putrefaction.

On Thursday, July 4, Dr. Gomez's paper was finished. He made comparative experiments on the disinfecting powers of vinegar, chlorine, and the fumes of sulphur. All of these substances destroyed completely the smell of putridity. With vinegar, it was necessary to dip the letters in the liquid, and then dry them slowly by heat. It was necessary to continue the fumes of chlorine till the writing on the back of the letter was somewhat injured. But the fumes of burning sulphur, mixed with nitre, destroyed the smell completely, without in the least injuring the writing, and more promptly than the other two methods. He considers it, therefore, as the best method of disinfecting letters, to expose them to the fumes of burning sulphur, mixed with saltpetre.

---

*Royal College of Surgeons.—Jacksonian Prize.*—The prize subject, for the year 1817, for the premium of 10l. to the author of the best Dissertation on a practical subject in Surgery, is "Deafness, and the Diseases and Injuries of the Organ of Hearing." Candidates must be members of the College, not on the Court of Assistants. The prize subjects for the present year, 1816, are, "Scrofula" and "Syphilis;" Dissertations upon which must be delivered before Christmas-day next.

---

*Prize proposed by the Royal Medical Society of Edinburgh.*—"What are the chemical changes produced in the air by the growth of plants; and do they on the whole purify or deteriorate the atmosphere? A set of books, or a medal of five guineas value, shall be given annually to the author of the best Dissertation on an experimental subject proposed by the Society; for which all the members, honorary, extraordinary, and ordinary, shall alone be invited as candidates. The Dissertations are to be written in English, French, or Latin, and to be delivered to the secretary on or before the 1st of December of the succeeding year.

Dr. Gordon communicated observations to prove that the appearance called the *buffy coat* or *inflammatory* crust is occasionally seen on arterial as well as on venous blood; also observations on the muscles of the living human body during surgical operations—on muscles of limbs immediately after amputation—and on the muscles of some of the lower animals. The result is, that the muscular fibre during its contraction does not exhibit any appearance of rugæ, but remains straight; and is not perceptibly enlarged in its transverse diameter.

Mr. Brooks's Museum is enriched by a human skull, whose appearance is unique, and the disease attacking both jaws different from any thing which has yet appeared in either human or brute osteology. The whole of the lower jaw, excepting the processes and articulation, is covered with a porous excrescence, resembling a reddisha reddish-brown spongue, but quite firm in its texture. The same kind of excrescence fills up the hollow on the sides of the nose and below it to the alveolar processes. The skull has been sent from Lima in South America. The history of the subject is entirely unknown, nor has any conjecture been hitherto formed of the nature of the disease, which is quite different from the bony shell formed round the sequestra in Necrosis.

---

*Crystallization of Lime.*—GAY-LUSSAC has lately ascertained that pure lime crystallizes in regular six-sided prisms. His method of proceeding was very ingenious. He put a quantity of lime-water into an open glass vessel, which he enclosed, together with another open vessel, containing strong sulphuric acid, in the exhausted receiver of an air-pump. When the sulphuric acid became too weak to act powerfully on the aqueous vapour, it was renewed. By this means the lime-water was made to evaporate pretty rapidly, and fragments of regular six-sided prisms were deposited in abundance. He gives a short account of these experiments in the Annales de Chimie and Physique for March, just published.

---

*On the Preservation of the Teeth;* by M. DUVAL, Surgeon-dentist, of Paris.

Many persons pretend that the teeth ought never to be filed; they imagine, first, that the teeth are loosened thereby, and apt to fall out; secondly, that the enamel being partly destroyed, they imbibe the saliva and humidity, more easily soften, and are consequently more apt to become carious. These persons, therefore, refuse to submit to an operation in itself very simple, and by means of which we may frequently preserve those organs, without which beauty itself is nothing, and the good state of which is absolutely necessary to health. These considerations are too important to suffer such prejudices to go uncorrected.

First, The teeth are enchased in their alveoles in such a manner that the file properly handled cannot loosen them, the force employed being far inferior to that exerted in the common mastication of food, without mentioning the shocks to which they are frequently exposed. When extreme sensibility of nerve renders the action of the file painful, the able dentist will easily devise means of rendering the operation more supportable.

Secondly, It is not true, that a carious tooth from which the enamel has been removed, is more liable to decay than the others; the bony substance exposed to the air becomes, on the contrary, drier and harder, and in a little time it loses the sensibility which rendered it accessible to cold or acids. Many persons preserve their teeth a great number of years after being filed, without experiencing the slightest inconvenience.

It is not only to stop the progress of caries that the teeth ought to be filed, it ought to be had recourse to, in order to remedy the original deformities of the teeth; it sometimes too removes the tooth-ache, ache, or fixes the teeth firmer in their place.* This custom is of the highest antiquity. Saint Jerome had his teeth filed to speak Hebrew with greater facility; undoubtedly some irregularity prevented his pronouncing correctly.

The savage nations have very ridiculous customs in this respect; some file the incisores to a point, others file them in two in the middle so as to double the number. All these examples prove, that, whenever this operation may be of service, it may be employed without any apprehension.

*Editors' Remarks.*—Notwithstanding it is now many years since the attention of anatomists was directed to the teeth by Mr. Hunter, and that the beneficial results of his exertions are every day seen and acknowledged, it is not less evident, that much good might frequently be done, both to adults and children, could the prejudices which unfortunately influence them be overcome.

M. Duval's remarks have considerable merit. In our opinion, he asserts too generally that filing teeth, taking away enamel, &c. can never be prejudicial, but, on the contrary, must invariably be attended with benefit. To support this opinion, he adds, that the soft bone of teeth, when exposed, not only loses its sensibility, but absolutely becomes "drier and harder" than before such denudation took place;—Why did he omit remarking, that in process of time it becomes black and carious?

If his remarks do not argue an indiscriminate use of the file, and will only urge the professional man to a little bolder practice than his deference to the prejudices of his patients has yet allowed, they may not prove injurious, but we have witnessed many injuries from the indiscriminate use of destructive instruments.

On subjects, however, more advanced, much care is necessary in estimating the proportion of benefit likely to result from the operation when decided upon; a file should never be used of greater thickness than a common card, and if there is no spot of discoloured enamel, a file serrated on both sides will be best; by which means, a thin layer of enamel will be taken off each tooth, and no particle of soft bone exposed; on the contrary, where the pressure has been great enough to produce a fracture of the enamel of one tooth, a file with a singly serrated side and edge will be better by taking off the whole thickness of the file from the diseased tooth.

A theory has of late been advanced, that the diseased matter of the bone of teeth acts similarly to the specific matter of cancer, that it has the property of disseminating its diseased principles; and that, by entirely extirpating every particle of black and brown bone, the disease will cease, and the remaining part of the tooth

* It is to be observed, that, when the tooth-ache is occasioned by the pressure of one tooth against another from their malconformation, the pain will be in the tooth pressed upon, and, therefore, to remove the cause of the pain, it is necessary to file the irregular tooth until it ceases to press.

 immediately immediately become secured, and will remain as sound as any other in which no disease has commenced.

This opinion has been founded upon the fact, that the teeth of adults, perfectly formed and fully ossified, may be filed and cut to almost any extent in subjects from eleven to sixteen years old. The teeth are but very imperfectly formed, the pulps are extremely large and correspondingly sensible; in these cases, were the whole of the discoloured matter to be picked out, the distress excited would compel the patient to loose the tooth.

In a future Number we shall offer some further remarks on a branch of surgery scarcely less important to comfort than to beauty, and on a part of the human growth which it becomes every practitioner, remote from the metropolis, carefully to watch in the younger branches of the families they are in the habit of visiting.

---

The following gentlemen have obtained the degree of Doctor in Medicine from the University of Glasgow, within the last twelve months:—Messrs. Thomas Nelson, Morrice Alexander, George Montgomery, and Archibald Lang, from Scotland; John Johnston, and Christopher Thornton, from Ireland; Andrew Dick, from Scotland; William Owens Allen, from Ireland.

---

Dr. John Squire, of Ely-place, at the age of 82, suddenly finished his long life of usefulness, at the house of a patient. This truly respectable veteran in physic began his career as a general practitioner in Wandsworth, where he practised, with much reputation, between twenty and thirty years. Though assisted, during that period, with partners, at different times, the labour was still greater than compensated by remuneration. Advancing to his 50th year, he embraced the obstetric practice, in the metropolis; and was very soon chosen physician to the charity for Delivering Lying-in Women at their own Houses. This institution furnished him with the advantage of so many cases, as to render his lectures extremely useful, in a branch of the profession in which nothing but practice can be of much use to the student. All his hearers had as many ordinary labours as they chose to attend; and the number of preternatural cases was greater than any other teacher could exhibit. By these advantages he retained a class long after the powers of eloquence had left him. His practice was a good deal, though not entirely, local, and very much in that class whose payment was unequal to the fatigues he must have submitted to; but these were greatly lessened, and his usefulness increased, by propinquity. He retained his health and faculties to the last, excepting that his hearing was somewhat dull. He has left a considerable family, all of whom do credit to his memory; but, it is painful to recollect, that the fortune of war deprived him of his great pride, Major Squire, of the engineers, who, after feasting his sire, then *narrative by age*, with honourable mention from his commander in most of the severe engagements in Spain, at last met with an honourable death in that unfortunate country, the grave of so many brave Britons, who fancied themselves fighting in the cause of freedom and true religion.

---

Mr. SALISBURY'S *Botanical Excursions, August* 1816.

August 3d.—Highgate.

Carex vulpina.
Carduus nutans.
Anthemis Cotula.
Atriplex erecta.
Agrostis alba.
——— spica venti.
Burtsia odentites.
Anthemis arvensis.
Chenopodium murale.
Brassica Napus.
Erica vulgaris.
Hypericum perforatum.
Carduus acanthoides.
Tamus communis.
Lotus corniculatus (var hirsutus)
Bromus asper.
——— giganteus.
Epilobium hirsutum.
Hypericum hirsutum.
Lysimachia Num mularia.
Rosa arvensis.
Geum arbanum.
Trifolium officinale.
Epilobium parviflorum.
Tragapogon porrifolium.
Medicago sativa.
Matricaria Parthenium.

August 10th.—Wimbledon Common and Barnes.

Poa rigida.
Fumaria claviculata.
Chelidonum majus.
Valeriana officinalis.
Medicago lupulina.
Leontodon autumnale.
Trifolium filiform.
Gnaphalium gallicum.
Peplis Portula.
Scirpus fluitans.
Pedicularis sylvaticus.
Juncus articulatus.
Potentilla verna.
Teucrium scorodonum.
Lychnis dioica v. alba.
Gnaphalium germanicum.
Onoris fruticosa.
Juncus bufonius.
——— uliginosus.
——— squarrosus.
Genista anglica.
Radiola millegrana.
Melica cœrulea.
Cuscuta europœa.
Festuca decumbens.
Gnaphalium rectum.
——— minimum.
Parietaria officinalis.
Menyanthes trifoliata.
Hydrocotyle vulgaris.
Rhamnus Catharticus.
Jasione montana.
Agrostis setacea.
Hypericum humifusum.
Cerastium semidicandrium.
Inula dysenterica.
——— pulicaria.
Cynoglossum officinale.
Senecio sylvatica.
——— erucæfolius.
——— Jacobœa.
Galeopsis tetrahit.
——— villosa.
Rosa spinosissima.
Trifolium glomeratum.
Galeopsis Ladanum.
Lythrum salicaria.
Hypericum quadrangulare.
Scrophularia aquatica.
Epilobium villosum.
——— palustre.
Ceratophyllum demersum.
——— submersum.

August 14th.—Hyde Park and Bayswater.

Poa Cristata.
Chenopodium Bonus Henricus.
Myriophyllum spicatum.
Hydrocotyle inundata.
Euphorbia Paralias.
Elynus caninus.
——— arenarius.
Mentha rotundifolia.
——— villosa.
——— sylvestris.

Mentha

Mentha Pulegium.
——— arvensis.
Nymphæa lutea.
——— alba.
Matricaria Chamomilla.
Phleum paniculatum.
Phalaris canariensis.
——— arundinacea.
Plantago media.
Polemonium cæruleum.
Phalaris arundinacea.
Prenanthes muralis.
Pastinaca sylvestris.
Pimpinella Saxifraga.
Picris hieraciodes.
Poterium Sanguisorba.
Sagittaria Sagittifolia.
Stipa pinnata (garden)
Sambucus Ebulus.
Statice armeria.
Saponaria officinalis.
Sedum anglicum.
——— Telephium.
——— dasyphyllum.
Sempervivum tectorum.
Antirrhinum spurium.
Angelica sylvestris.
Allium Vineale.
Agrimonia Eupatoria.
Artemisia vulgaris.
Atriplex patula.
Asplenium Ruta muraria.
Bromus squarrosus.
Cerastium aquaticum.
Centaurea Calcitrapa.
——— scabiosa.
Dipsacus sylvestris.
Datura stramonium.
Erigeron canadense.
Leonurus Cardiaca.
Melissa Nepeta.
Origanum onites.
Osmunda spicant.
Poa aquatica.
Peucedanum Silaus.
Senecio sarracenicus.
Sium nodiflorum.
——— angustifolium.
Veronica scutellata.

August 21st.—Battersea Fields and Wandsworth Common.

Apium graveolens.
Avena fatua.
Atropa Belladonna.
Anethum fæniculum.
Althæa officinalis.
Arctium Lappa.
Briza media.
Bromus secalinus.
——— diandrus.
——— hirsutus.
——— sylvaticus.
Butomus umbellatus.
Betonica officinalis.
Brassica tenuifolia.
Campanula Rapunculus.
Cuscuta europœa.
Caucalis nodosa.
——— latifolia.
——— daucoides.
——— infesta.
Crepis tectorum.
——— biennis.
Circæa lutetiana.
Convolvulus sepium.
Chironia Centaureum.
Cheropodium olidum.
——— ficifolium.
——— hybridum.
——— urbicum.
Conium maculatum.
Coriandrum sativum.
Cistus Helianthenum.
Clinopodium vulgare.
Cichorium Intybus.
Epilobium palustre.
Erysinum chiranthoides.
Galium Mollugo.
Gnaphalium margarataceum.
Geranium sylvaticum.
Heracleum sphondylium.
Hyosciamus niger.
Lycopus europæus.
Lolium temulentum.
Linum usitatissimum.
Lysimachia thyrsiflora.
Lepidium latifolium.
Lactuca scariola.
——— virosa.
Leontodon palustre.
Zannichellia palustris.

August 24th.—Wimbledon Common and Woods.

Trifolium scabrum.

——— arvense.

Gnaphalium margaritaceum.

Rubus corylifolius.

——— cæsius.

Hieracium sylvaticum.

| | |
|---|---|
| Inula dysenterica. | Centaurea Calcitrapa. |
| Clematis vitalba.' | Dipsacus pilosus. |
| Antirrhinum Orontium. | Inula pulicaria. |
| ——— Elatine. | Lysimachia vulgaris. |
| Artemisia vulgaris. | Marrubium vulgare. |
| Amaranthus Blitum.' | Scirpus acicularis. |
| Melampyrum sylvaticum. | Sison amomum. |
| Bidens tripartita.' | ——— segetum.' |
| ——— cernua.' | Stachys sylvatica. |

*Observations.*—I have heard it asserted, that, owing to the late rains, the corn, in general, is beginning to mildew, and to be otherwise diseased, a circumstance I have not observed near London. I do not think, on the whole, that there ever was a more abundant crop of all kinds of grain, and which, from the few hours of sunshine we have lately had, is getting ripe very fast. There is not any wheat yet cut. Some hay is still out, and most of it in very bad plight, owing to its being mown in wet weather, and no time since to get it up. There is, however, a fair prospect of an abundant after-math both of grass and clover.

*Botanic Garden, Sloane-street;* W. Salisbury.

*August* 25, 1816.

---

*Medical Theatre, St. Bartholomew's Hospital.*—The following Courses of Lectures will be delivered at this Theatre during the ensuing winter:—On the Theory and Practice of Medicine, by Dr. Hue. On Anatomy and Physiology, by Mr. Abernethy. On the Theory and Practice of Surgery, by ditto. On Chemistry and Materia Medica, by Dr. Hue. On Midwifery, by Dr. Gooch. The Demonstrations of Anatomy, by Mr. Stanley.—To commence on Tuesday, October 1st, at two o'clock.

*Medical School, St. Thomas's and Guy's Hospitals.*—The Winter Course of Lectures at these adjoining Hospitals will commence, as usual, the 1st of October, viz. at St. Thomas's: Anatomy, and the Operations of Surgery, by Mr. Astley Cooper and Mr. Henry Cline; Principles and Practice of Surgery, by Mr. Astley Cooper. At Guy's: Practice of Medicine, by Dr. Babington and Dr. Curry; Chemistry, by Dr. Babington, Dr. Marcet, and Mr. Allen; Experimental Philosophy, by Mr. Allen; Theory of Medicine, and Materia Medica, by Dr. Curry and Dr. Cholmeley; Midwifery, and Diseases of Women and Children, by Dr. Haighton; Physiology, or Laws of the Animal Œconomy, by ditto. These several lectures are so arranged, that no two of them interfere in the hours of attendance; and the whole is calculated to form a complete course of medical and chirurgical instruction.

*St. George's Medical, Chemical, and Chirurgical Schools.*—The Courses will commence the first week in October:—1st. On the Laws of the Animal Œconomy, and the Practice of Physic, by George Pearson, M.D. F.R.S. Senior Physician to St. George's Hospital, &c. 2d. On Therapeutics, with Materia Medica and Medical Jurisprudence, by George Pearson, M.D. and W. T. Brande, F.R.S. Professor, Royal Institution. 3d. On Chemistry, by by W. T. Brande, F.R.S. Professor of Chemistry, R. S. 4th. On the Theory and Practice of Surgery, by B. C. Brodie, F.R.S. Assistant-Surgeon to St. George's Hospital.—*Note.* Sir Everard Home continues to give Lectures on Surgery, gratuitously, to the pupils of St. George's Hospital.

The Medical Lectures in the University of Glasgow will begin on Tuesday, Nov. 5th, at the following hours:—Surgery, by Mr. Burns, at nine in the morning. Dietetics, Materia Medica, and Pharmacy, by Dr. Millar, at ten. Midwifery, by Mr. Towers, at eleven. Practice of Medicine, by Dr. Freer, at twelve. Anatomy, by Dr. Jeffray, at two in the afternoon. Institutions of Medicine, by Dr. Freer, at three. Chemistry, and Chemical Pharmacy, by Dr. Cleghorn, at seven.—Dr. Graham will commence his Lectures on Botany about the beginning of May next.

Dr. Adams will commence his Course of Lectures on the Institutes and Practice of Medicine early in October, at his house in Hatton Garden.

Dr. Clutterbuck will begin his Autumnal Course of Lectures on the Theory and Practice of Physic, Materia Medica, and Chemistry, on Wednesday, October 2d, at ten o'clock in the morning, at his house, No. 1, in the Crescent, New Bridge street, Blackfriars.

Dr. Collingwood, Physician to the Universal Medical Institution, will commence his Lectures on the Theory and Practice of Medicine, on Monday, Sept. 2, at eleven o'clock in the forenoon.

Mr. Wilson and Mr. Bell will commence their Lectures on Anatomy and Surgery, in Great Windmill-street, on the 1st of October, at two o'clock.—Mr. Bell will deliver a separate Course of Lectures on Surgery in the evening.—The Anatomical Demonstrations will be given by Mr. Shaw, who will direct the students in their dissections.

Mr. Clarke will commence his Winter Courses of Lectures on Midwifery, and the Diseases of Women and Children, on Thursday, October 3d, at a quarter-past ten in the morning.

In the first week in October, Dr. George Gilbert Currey, Fellow of the Royal College of Physicians, and Physician to St. Thomas's Hospital, will commence a Course of Lectures on the Practice and Theory of Medicine, and the Materia Medica.

---

### REPORT OF DISEASES.

The number of inflammatory diseases gradually lessens, but when they occur their severity is not greatly diminished; considering the distressed condition of the labouring class, this is enough to show, that the form of disease depends more, to use the language of Sydenham, on the constitution of the air than on the mode of life, though, without doubt, some modification must follow from those sources.

Several chronic complaints make their appearance, most of them the effect of neglect during previous acute paroxysms, from too tardy an application for relief, or too timid a practice in the medical attendant. They are still relieved, to a certain degree, by evacuations, chiefly topical; but, where the organic lesion is considerable, the relief is, of course, only partial. Thus, paralytic affections from extravasation or effusion on the brain, or spinal marrow, continue to keep the dispensaries employed; as well as abscesses, nodosities, or stiffness in the joints. Among children, inflammation of the brain, threatening effusion, and, if not prevented, ending in hydrocephalus, is more frequent or earlier suspected than ever. Does this arise from the foolish habit of mothers and wet nurses, thinking it right to swallow porter two or three times a day, in order, as they fancy, to increase their milk, or having recourse to a still more dangerous beverage whenever their appetite fails: whatever may be the cause, the malady is certainly very common in England, and, there is reason to believe, particularly in London and other large towns.

Towards the close of the month, catarrhs have been so frequent, though the weather has improved, that some practitioners have suspected an influenza. Something of this kind has appeared in America during the close of the past, and beginning of the present, year, of which we have the following account from Dr. Warren.

"About the time of the violent gale or hurricane of September 24, 1815, people began to complain of colds and sore throats; and, within a week after, an epidemic prevailed in Boston, which extended more generally than any before known. The invasion of this disease was often most sudden, the patient passing at once from a state of health to that of severe disease; in others, the symptoms of catarrh were its precursors. The symptoms were, chills followed by fever; pain, soreness and rigidity in the muscles, sometimes to a most distressing degree; nausea and vomiting; stupor; pulsating pain in the head, increased on motion; in bad cases, loss of sense; soreness and pain in the eyes; copious discharge of fluids from the nostrils; soreness of the throat; stricture and soreness across the breast; pain in the side of the breast, and dry painful cough. The violence of the symptoms usually abated in three days. Children were rarely affected; women less frequently than men; and the aged most dangerously. Of three fatal cases, two occurred in persons above sixty; the other in a labourer of forty, who had been peculiarly exposed, by sleeping all night on the ground in the open air. Many persons were left with weakness of the lungs, great susceptibility to catarrh, and disposition to phthisis pulmonalis.

"This complaint raged during a great part of the month of October; and at the beginning of November had nearly subsided. Instances occurred during the following winter. Soon after its appearance in Boston, it manifested itself throughout the northern and eastern states; thence travelled through the middle to the southern and western parts of the country, and appeared, as I am informed, in South Carolina in February."

If this kind of morbific atmosphere usually travels from east to west, as many physicians have remarked, and as has been proved in the present instance, we must expect to hear of it in our eastern settlements before it arrives amongst us.

### METEOROLOGICAL REGISTER.

***From July the 25th, to August the 25th,* 1816.**

Kept by C. BLUNT, Philosophical Instrument Maker, No. 38, Tavistock-Street, Covent-Garden.

| Moon. | Day. | Wind. | Barometrical Pressure. Max. | Min. | Mean. | Temperature. Max. | Min. | Mean. | |
|---|---|---|---|---|---|---|---|---|---|
| | 26 | W | 29·80 | 29·66 | 29·73 | 76 | 46 | 61· | Rain |
| | 27 | W | 30· | 30· | 30· | 69 | 48 | 58·5 | Rain |
| | 28 | N | 30· | 30· | 30· | 65 | 50 | 57·5 | Rain |
| | 29 | NE | 29 95 | 29·92 | 29·94 | 64 | 50 | 57· | Rain |
| | 30 | NE | 29·97 | 29·96 | 29 96 | 66 | 50 | 58· | Rain |
| ◐ | 31 | E | 30·01 | 30· | 30·00 | 67 | 47 | 57· | Rain |
| | 1 | E | 30 3 | 30·2 | 30·25 | 66 | 47 | 56 5 | Rain |
| | 2 | SE | 30·1 | 30· | 30·05 | 67 | 49 | 58· | Rain |
| | 3 | S | 30· | 29·95 | 29·97 | 69 | 50 | 59·5 | Rain |
| | 4 | S | 29·90 | 29·86 | 29·88 | 72 | 48 | 60· | Fair |
| | 5 | S | 29·90 | 29·80 | 29·85 | 71 | 49 | 60· | Rain |
| | 6 | S | 29·96 | 29·91 | 29·94 | 70 | 50 | 60· | Rain |
| | 7 | SW | 30·01 | 30· | 30·05 | 70 | 48 | 59· | Rain |
| ○ | 8 | W | 30· | 30· | 30· | 69 | 47 | 58· | Fair |
| | 9 | W | 30·20 | 30·16 | 30·18 | 69 | 45 | 57· | Rain |
| | 10 | NW | 30· | 30· | 30· | 62 | 49 | 55·5 | Fair |
| | 11 | SW | 30·10 | 30 06 | 30·08 | 70 | 50 | 60· | Fair |
| | 12 | SW | 30·10 | 30· | 30·05 | 70 | 51 | 60·5 | Rain |
| | 13 | S | 30· | 29·90 | 29·95 | 69 | 50 | 59 5 | Rain |
| | 14 | SW | 29 50 | 29·49 | 29.49 | 71 | 51 | 61· | Rain |
| | 15 | W | 29·62 | 29 49 | 29 55 | 68 | 50 | 59· | Rain |
| ◑ | 16 | SW | 29·83 | 29·82 | 29·82 | 68 | 50 | 59· | Rain |
| | 17 | S | 29·90 | 29·88 | 29·89 | 70 | 52 | 61· | Rain |
| | 18 | W | 30· | 29·90 | 29·95 | 62 | 49 | 55 5 | Rain |
| | 19 | NW | 30·20 | 30·10 | 30 15 | 65 | 49 | 57· | Rain |
| | 20 | NW | 30·35 | 30 32 | 30·33 | 64 | 45 | 55· | Fair |
| | 21 | N | 30·35 | 30·33 | 30·34 | 60 | 46 | 53· | Fair |
| | 22 | NW | 30·36 | 30·33 | 30 34 | 61 | 43 | 52· | Showers |
| ● | 23 | NW | 30·10 | 30· | 30·05 | 60 | 42 | 51· | Showers |
| | 24 | NW | 30·25 | 30·24 | 30·24 | 63 | 41 | 52· | Showers |
| | 25 | NW | 30·34 | 30·30 | 30 32 | 65 | 44 | 54·5 | Fair |

RESULTS.

| | | | |
|---|---|---|---|
| Mean barometrical pressure of the month | 31·95 | Mean temperature of the month | 57·67 deg. |
| Maximum 30·36, wind at | NW | Maximum 76, wind at | W |
| Minimum 29·49, ——— | W | Minimum 41, ——— | NW |

*Scale exhibiting the prevailing Winds during the Month.*

| N | NE | E | SE | S | SW | W | NW |
|---|---|---|---|---|---|---|---|
| 2 | 2 | 2 | 1 | 6 | 5 | 6 | 7 |

| | Mean barometrical pressure. | Mean temper |
|---|---|---|
| From the new moon on the 24th July, to the first quarter, on the 31st July | 29·99 | 57·94 |
| ——— the first quarter on the 31st, to the full moon on the 8th of August | 29·99 | 58 87 |
| ——— the full moon on the 8th, to the last quarter on the 16th | 29·89 | 58 93 |
| ——— the last quarter on the 16th, to the new moon on the 23d | 30·15 | 54 93 |

CATALOGUE

